# Altered gut bacterial–fungal interkingdom networks in patients with current depressive episode

**DOI:** 10.1002/brb3.1677

**Published:** 2020-06-12

**Authors:** Hai‐yin Jiang, Li‐ya Pan, Xue Zhang, Zhe Zhang, Yuan‐yue Zhou, Bing Ruan

**Affiliations:** ^1^ Collaborative Innovation Center for Diagnosis and Treatment of Infectious Diseases State Key Laboratory for Diagnosis and Treatment of Infectious Diseases the First Affiliated Hospital School of Medicine Zhejiang University Hangzhou China; ^2^ Department of Urology The First Affiliated Hospital College of Medicine Zhejiang University Hangzhou China; ^3^ Department of Child Psychiatry Hangzhou Seventh People’s Hospital Hangzhou China

**Keywords:** dysbiosis, fungi, gut microbiota–brain, mental

## Abstract

**Introduction:**

Bacterial dysbiosis has been described in patients with current depressive episode (CDE); however, the fungal composition in the gut has not been investigated in these patients.

**Methods:**

Here, we characterized the fungal gut mycobiota in patients with CDE. We systematically characterized the microbiota and mycobiota in fecal samples obtained from 24 patients with CDE and 16 healthy controls (HC) using 16S rRNA gene‐ and ITS1‐based DNA sequencing, respectively.

**Results:**

In patients with CDE, bacterial dysbiosis was characterized by an altered composition and reduced correlation network density, and the gut mycobiota was characterized by a relative reduction in alpha diversity and altered composition. Most notably, the CDE group had higher levels of Candida and lower level of Penicillium than the HC group. Compared with the HC group, the gut microbiota in patients with CDE displayed a significant disruption in the bacteria–fungi correlation network suggestive of altered interkingdom interactions. Furthermore, a gut microbial index based on the combination of eight genera (four bacterial and four fungal CDE‐associated genera) distinguished CDE patients from controls with an area under the curve of approximately 0.84, suggesting that the gut microbiome signature is a promising tool for disease classification.

**Conclusions:**

Our findings suggest that both bacteria and fungi contribute to gut dysbiosis in patients with CDE. Future studies involving larger cohorts and metagenomic or metabolomic analyses may clarify the structure and potential roles and functions of the gut mycobiome and its impact on the development of CDE.

## Graphical abstract

Patients with current depressive episode (CDE) display bacterial and fungal gut dysbiosis. Fungal gut dysbiosis in CDE is characterized by an altered composition and reduced biodiversity. In patients with CDE, the correlation network between bacteria and fungi is disrupted. Our findings suggest that the gut mycobiota contributes to the pathogenesis of CDE and should be considered as a new target for therapeutic intervention.

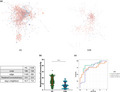

## 1 INTRODUCTION

The recent proposal of a gut microbiota–brain axis has stimulated intense interest in the composition of the bacterial microbiota in the gut and related dysbiosis in patients with psychiatric disorders (Bailey & Cryan, 2017; Foster & McVey Neufeld, 2013). In contrast, the fungal constituents of the microbiome, known as the mycobiome, have received little attention despite being an important component of the gut microbiome. Although the number of fungi in the gut is significantly lower than that of commensal bacteria, emerging evidence has underscored the importance of fungi in host health and microbe–microbe interactions (Nash et al., 2017). Thus, investigation of the gut mycobiome and its relationship with the bacterial members of the microbiota in patients with affective disorders would increase our understanding of the underlying mechanisms and role of the gut mycobiota in the development of psychiatric conditions.

Several studies have found close relationships between fungal infections and psychiatric diseases. *Candida albicans* antibody level is elevated in patients with schizophrenia and is associated with worse psychiatric symptoms (Severance et al., 2016). Probiotics have been shown to reduce *C. albicans* antibody levels and relieve yeast‐related bowel discomfort, implicating altered host‐associated fungal populations in the development of psychiatric diseases (Severance et al., 2017). An understanding of the mycobial diversity and abundance in psychiatric disorders is essential for clarifying the role of fungal species in the gut microbiota–brain axis.

Over the past decade, accumulating evidence showed that gut microbiota is essential for brain function, namely through inflammation, and the hypothalamic–pituitary–adrenal (HPA) axis, and by affecting neurotransmission (Foster & McVey Neufeld, 2013). Although the pathways linking gut bacteria with the brain are incompletely understood, an emerging body of research is beginning to link the intestinal bacterial microbiota with current depressive episode (CDE). Gut microbiota changes associated with depression include a decrease in some species of the phylum Firmicutes and an increase in species of the phylum Proteobacteria (Jiang et al., 2015; Kelly et al., 2016). Several recent studies have shown alterations in the gut microbiota of patients with bipolar depression, including decreased biodiversity, a decrease in Firmicutes species (e.g., Faecalibacterium), and an increase in Proteobacteria species (e.g., Enterobacteriaceae; Coello et al., 2019; Jiang, Xu, Zhang, Zhang, & Ruan, 2019). Thus, dysbiosis in CDE was characterized by a predominance of some potentially harmful bacterial groups and a reduction in beneficial bacterial genera. To date, all investigations of the gut microbiome in patients with CDE have focused on bacterial dysbiosis, whereas changes in the fungal mycobiota have not been studied. We characterized the bacterial gut microbiota and mycobiota in patients with CDE through an amplicon‐based metataxonomic analysis of the V3–V4 regions of the prokaryotic 16S ribosomal DNA (rDNA) and the internal transcribed spacer (ITS)1 region of the fungal rDNA to better understand the microbial community structure associated with the depressive state.

## 2 METHODS

### 2.1 Subject selection

This study protocol was approved by the Ethics Committee of The First Affiliated Hospital of Zhejiang University. Our study included 24 patients with CDE from the Seventh People's Hospital of Hangzhou and 16 healthy control subjects (HC) recruited from the First Affiliated Hospital of Zhejiang University. All participants were screened by an experienced psychiatrist using the Mini‐International Neuropsychiatric Interview (Sheehan et al., 1998). Bipolar disorder (BD) and depression were diagnosed according to the criteria of the Structured Clinical Interview for the Diagnostic and Statistical Manual of Mental Disorders‐Fourth Edition (First, Spitzer, Gibbon, & Williams, 1996). The severity of depressive symptoms was assessed using the 24‐item clinician‐administered Hamilton Depression Scale (Hamilton, 1959). All patients with CDE had been hospitalized and were examined clinically before sampling. After informed consent was obtained, the patients were interviewed about drug use within 4 weeks of admission by specially trained nurses using a structured questionnaire.

### 2.2 Fecal sample collection and DNA extraction

Prior to the collection of the fecal samples, none of the subjects had taken antibiotics drugs or probiotic, prebiotic, or symbiotic supplements in the previous 2 months. Obese individuals and those with diabetes, cardiovascular disease, irritable bowel syndrome, inflammatory bowel disease, autoimmune diseases, allergic diseases, or other comorbidities; those who had abused drugs or alcohol in the last year; and those who had known active bacterial, fungal, or viral infections were excluded from the study. Also, patients had a history of gastrointestinal surgery and accepted colonoscopy before 4 weeks ago were not included in our studies.

Fresh fecal samples were collected in a sterile plastic cup at the hospital and refrigerated. The samples for bacterial genomic DNA extraction were delivered to the laboratory within 30 min of collection and stored at −80°C. DNA extractions were performed using a FastDNATM SPIN Kit for Feces (MP Biomedicals) according to the manufacturer's protocol with additional glass‐bead beating steps performed using a Mini‐Beadbeater (Thermo Electron Corp.). DNA was quantified using a NanoDrop ND‐2000 spectrophotometer (Thermo Electron Corp.), and the integrity and size were assessed using 1.0% agarose gel electrophoresis on gels containing 0.5 mg/ml of ethidium bromide. Finally, DNA was stored at −20°C until further use.

### 2.3 Polymerase chain reaction (PCR) analysis and pyrosequencing

The genomic DNA isolated from the clinical samples was amplified using the PCR procedure using the barcode primer set 338F ACTCCTACGGGAGGCAGCAG and 806R GGACTACHVGGGTWTCTAAT, and the ITS regions using the primer set ITS1F CTTGGTCATTTAGAGGAAGTAA and ITS2R GCTGCGTTCTTCATCGATGC, respectively. The amplicons were then purified using gel extraction and quantified using QuantiFluor‐ST (Promega Corp.). The purified amplicons were pooled in equimolar concentrations and sequenced using the Illumina MiSeq platform with a TruSeq™ DNA Sample Prep Kit (Illumina).

### 2.4 Bioinformatics and statistical analyses

The sequencing reads were processed using Quantitative Insights into Microbial Ecology software. Preliminary quality control steps were performed as described previously (Caporaso et al., 2010; Jiang et al., 2018), and chimeric sequences were removed using ChimeraSlayer. The remaining effective sequences were binned into operational taxonomic units (OTUs) with a cutoff of 97% identity to determine alpha diversity (Shannon, Simpson, ACE, and Chao indices). Beta diversity was estimated by calculating the Bray‐Curtis or the unweighted or weighted UniFrac distance, and visualized using principal coordinate analysis (PCoA). Bacterial or fungal genera with an average relative abundance of ≥0.01 in samples were considered to be major genera. A linear discriminant analysis effect size (LEfSe) procedure was used to identify microbiome biomarkers in the fecal samples of the CDE patients and HC at various taxonomic ranks. The bacterial and fungal groups with a linear discriminant analysis (LDA) score of 2 were defined as significantly abundant (Segata et al., 2011). The relative connectedness of the networks was calculated as the ratio between the number of significant interactions (edges) and the number of taxa (nodes) in the network. All statistical tests were performed using SPSS statistical software (version 21.0; IBM Corp.) or R package (version 2.15.3; McMurdie & Holmes, 2013). All tests for significance were two‐sided, and *p*‐values <.05 were considered to indicate statistical significance.

### 2.5 Accession numbers

The sequence data from this study have been deposited in the GenBank Sequence Read Archive with the accession number SRP221419.

## 3 RESULTS

### 3.1 Participant characteristics

We recruited 24 patients with CDE (BD II, *n* = 10; major depressive disorder, *n* = 14) and 16 unrelated HC. The demographic and clinical characteristics of the participants are shown in Table 1. No significant differences in age, sex, or body mass index (BMI) were found between the CDE group and HC. The mean total HAMD score was 25.6, indicating that most of the patients with severe symptoms required medical treatment.

**TABLE 1:** Demographic characteristics of study subjects

Parameters	Healthy controls (HC) *n* = 16	Patients with Current Depressive Episode (CDE) *n* = 24	*p* value
Proportion of Females, No. (%)	7 (43.8%)	11 (45.8%)	>.05
Age (years; means ± *SD*) (range)	35.8 ± 6.8	37.2 ± 7.2	>.05
BMI (means ± *SD*) (range)	22.3 ± 6.5	23.6 ± 7.1	>.05
Smoking, No. (%)	1 (6.3%)	2 (8.3%)	>.05
Psychiatric family history, No. (%)	0	2 (8.3%)	–
Antidepressant, No. (%)	0	11 (45.8%)	–
Benzodiazepines, No. (%)	0	16 (66.7%)	–
Atypical antipsychotic, No. (%)	0	9 (37.5%)	–
Mood stabilizer, No. (%)	0	7 (29.2%)	–
HAMDS	2.3 ± 1.1	25.3 ± 6.8	–

### 3.2 Bacterial dysbiosis in CDE

**FIGURE 1:**
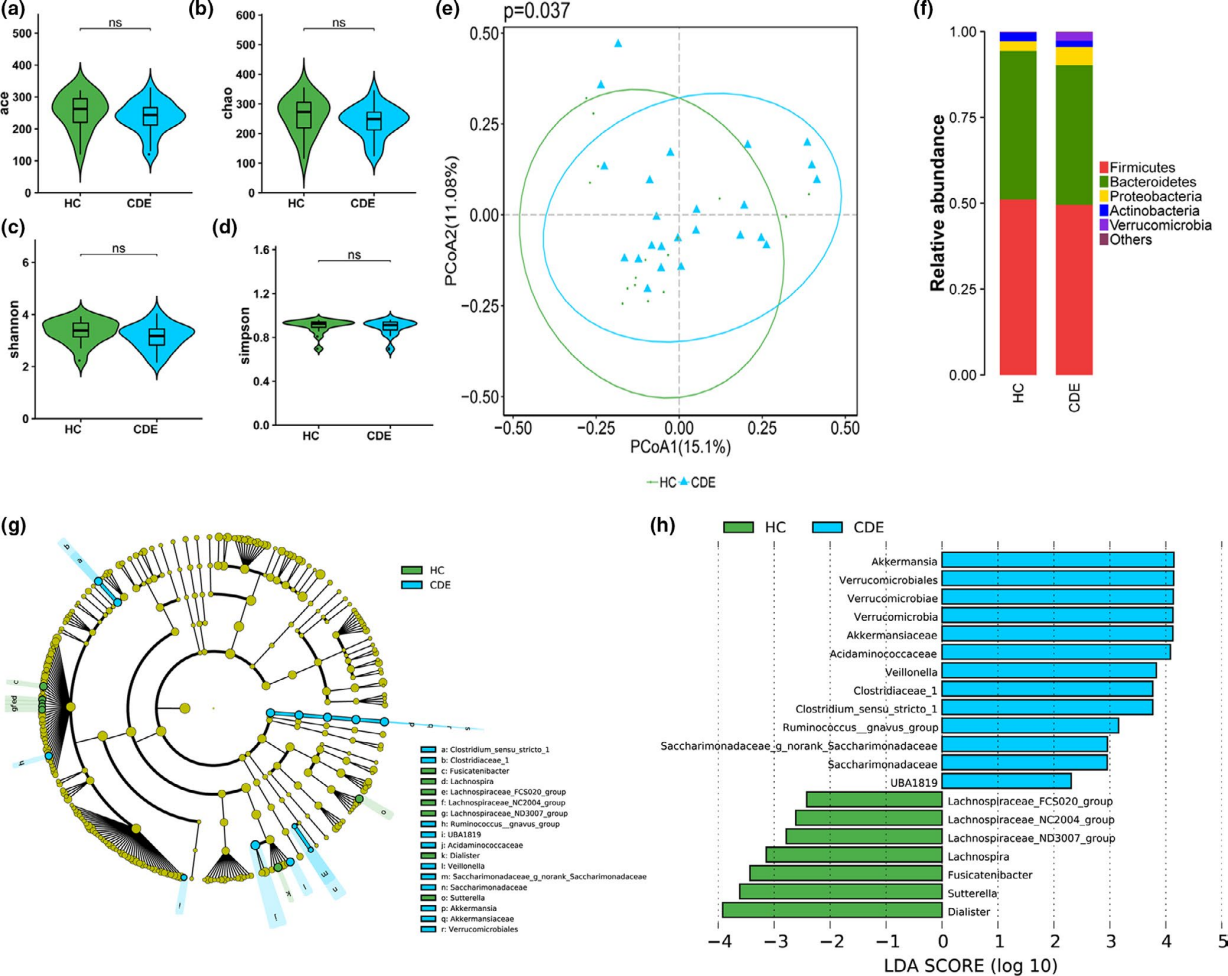
Phylogenetic diversity of gut bacterial microbiomes among CDE and HC. Comparison of the ace (a), Chao (b), shannon (c), and simpson (d) index between two groups; (e) PCoA of Bray‐Curtis distance matrix analysis demonstrated that the bacterial microbiome composition of CDE clustered separately from HC; (f) Composition of fecal bacterial microbiome at the phylum level between the two groups; (g) Taxonomic cladogram obtained from LEfSe analysis of 16S sequences. (Blue) CDE taxa; (Green) taxa enriched in HCs. The brightness of each dot is proportional to its effect size. (h) HC‐enriched taxa are indicated with a positive LDA score (Green), and taxa enriched in CDE have a negative score (Blue). Only taxa meeting an LDA significant threshold > 2 are shown. * indicates *p* < .05, ns indicates *p* > .05

The bacterial fraction of the gut microbiota was characterized using high‐throughput sequencing of the bacterial 16S rRNA gene. The alpha diversity, as determined by the mean ACE, Chao, Shannon, and Simpson reciprocal indexes, was not significantly different between groups (Figure 1a–d). Beta diversity analysis performed using the Bray‐Curtis distance matrix and PCoA plots revealed significant clustering between the HC and CDE group (Figure 1e). However, the PCoA of the unweighted and weighted UniFrac distances did not discriminate between the HC and CDE group (Figures S1 and S2). To evaluate the influence of CDE type on the gut bacterial composition, we further analyze the difference between BD II and depression patients. No significant differences were observed in α‐diversity between two groups, as indicated by the ACE, Chao, Shannon, and Simpson indices (Table S1 and Figures S3–S5). Hierarchical clustering on Bray‐Curtis, Unweighted, and Weighted UniFrac cannot separate BD II patients from depression patients. We next examined whether antidepressant use was associated with the gut bacterial microbiome. No significant difference was observed in the α‐diversity or β‐diversity between antidepressant users and non‐users (Table S1 and Figures S6–S8). We then classified the patients according to antipsychotic use. However, neither within‐individual nor between‐individual diversity was significantly related to antipsychotic exposure in our cohort (Table S1 and Figures S9–S11).

Overall microbial composition analysis revealed that the bacterial gut microbiota was dominated by species from the phyla Firmicutes, Bacteroidetes, Actinobacteria, and Proteobacteria (Figure 1f). To identify differentially abundant taxa, we performed an LEfSe analysis on the fecal microbiota of the CDE group and HC (Figure 1g,h). At the genus level, significant differences were observed between the CDE group and HC: 10 bacterial taxa showed distinct relative abundances between groups (LDA score > 2.0, *p* < .05). Decreased abundance in bacteria, including *Dialister*, *Fusicatenibacter*, *Lachnospira*, *Lachnospiraceae_FCS020_group*, *Lachnospiraceae_NC2004_group*, *Lachnospiraceae_ND3007_group*, and *Sutterella*, and increased abundance in *Akkermansia*, *Clostridium_sensu_stricto_1*, *UBA1819*, and *Veillonella* were observed in the patients with CDE.

### 3.3 Fungal dysbiosis in CDE

**FIGURE 2:**
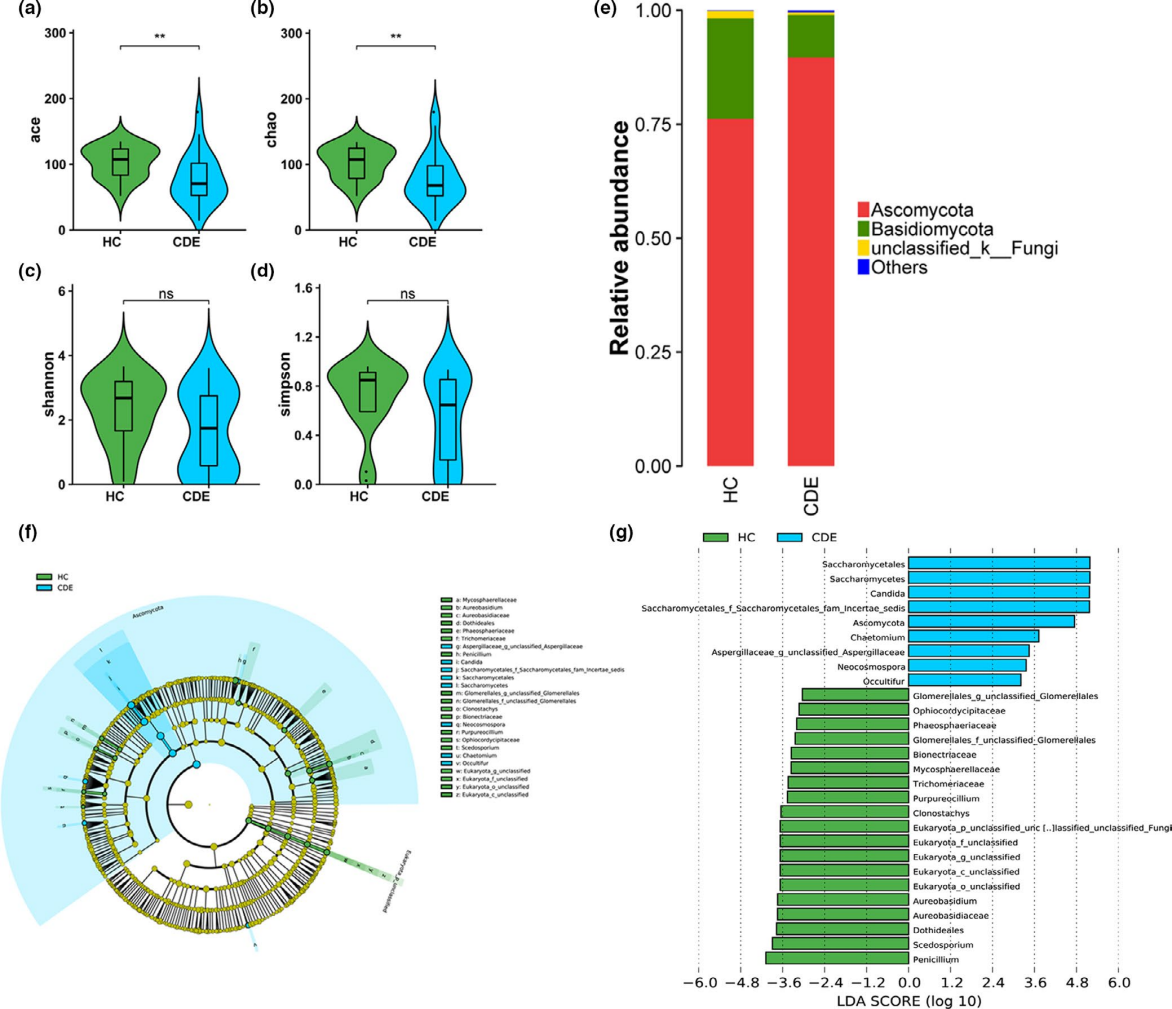
Phylogenetic diversity of gut fungal microbiomes among CDE and HC. Comparison of the ace (a), Chao (b), shannon (c), and simpson (d) index between two groups; (e) Composition of fecal fungal microbiome at the phylum level between the two groups; (f) Taxonomic cladogram obtained from LEfSe analysis of ITS sequences. (Blue) CDE taxa; (Green) taxa enriched in HCs. The brightness of each dot is proportional to its effect size. (g) HC‐enriched taxa are indicated with a positive LDA score (Green), and taxa enriched in CDE have a negative score (Blue). Only taxa meeting an LDA significant threshold >2 are shown. * *p* < .05, ** indicates *p* < .01, ns indicates *p* > .05

The gut mycobiota of our study cohort was characterized using amplicon‐based sequencing of the fungal ITS1 region. The alpha diversity (assessed using the ACE and Chao indices) of the gut mycobiota in patients with CDE was reduced relative to that of the HC, whereas the Shannon and Simpson indexes were not significantly different between the groups (Figure 2a–d). PCoA of beta diversity based on the Bray‐Curtis and unweighted or weighted UniFrac distance did not separate the CDE and control samples (Figures S12–S14). To evaluate the influence of CDE type on the gut fungal composition, we further analyze the difference between BD II and depression patients. No significant difference was observed in α‐diversity between two groups, as indicated by the ACE, Chao, Shannon, and Simpson indices. Hierarchical clustering on Bray‐Curtis, Unweighted, and Weighted UniFrac cannot separate BD II patients from depression patients (Table S2 and Figures S15–S17). We next examined whether antidepressant use was associated with the gut fungal microbiome. No significant difference was observed in the α‐diversity or β‐diversity between antidepressant users and non‐users (Table S2 and Figures S18–S20). We then classified the patients according to antipsychotic use. However, neither within‐individual nor between‐individual diversity was significantly related to antipsychotic exposure in our cohort (Table S2 and Figures S21–S23).

The fecal fungal microbiota in both groups was dominated by the phyla Basidiomycota and Ascomycota, accounting for 98% of fecal samples (Figure 2e). LEfSe analysis revealed several differences between the CDE participants and HC (Figure 2f,g). At the genus level, 12 fungal taxa showed distinct relative abundances between groups (LDA score > 2.0, *p* < .05): *Candida*, *Chaetomium*, *Neocosmospora*, *Occultifur*, *Neocosmospora*, *Clonostachys*, and *Chaetomium* were overrepresented in patients with CDE compared with the HC, whereas *Scedosporium*, *Purpureocillium*, *Penicillium*, *Clonostachys*, and *Aureobasidium* were decreased relative to the HC.

### 3.4 An altered interkingdom network in CDE

**FIGURE 3:**
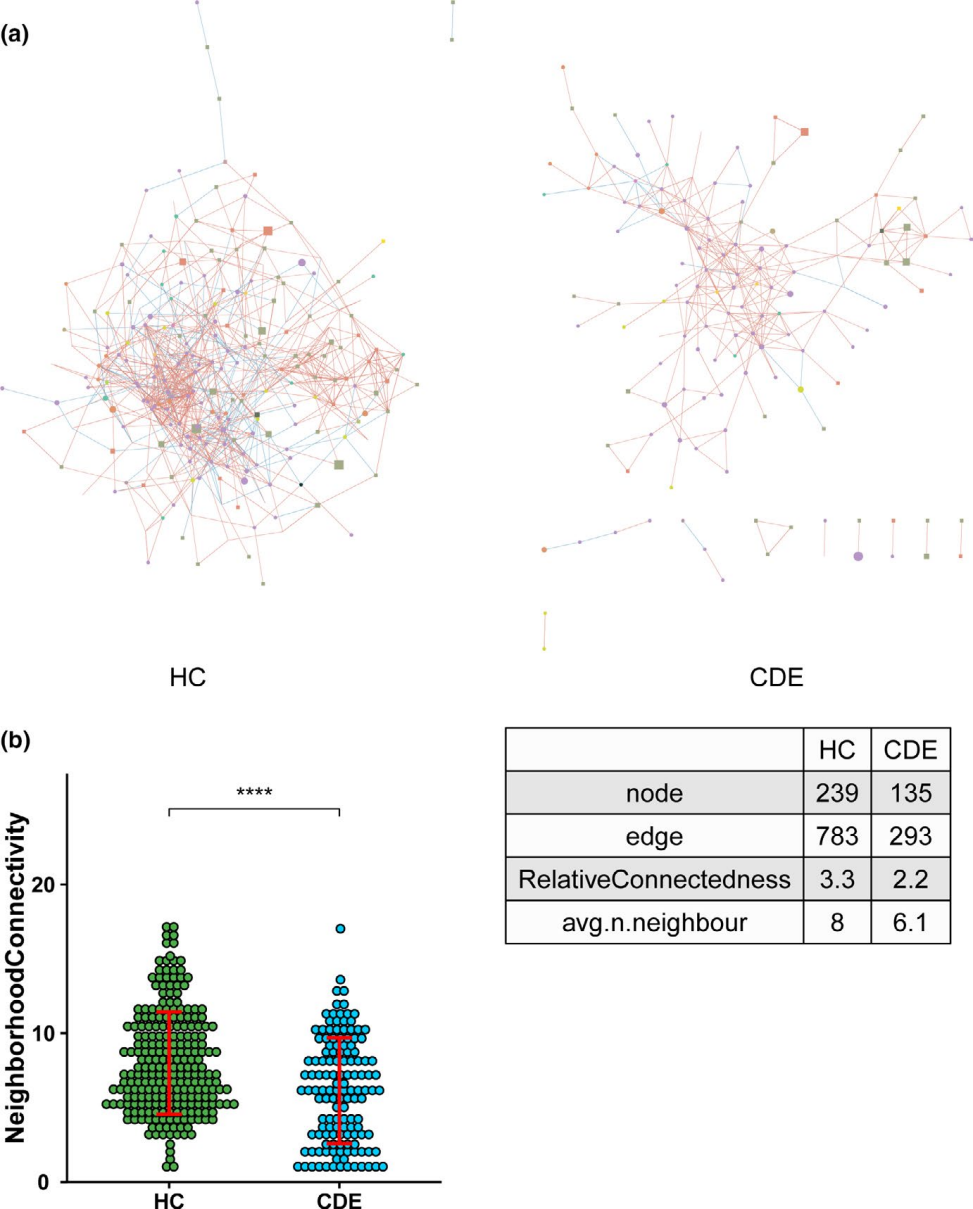
(a) Correlation networks between bacteria were built using Spearman's correlation test in the two study groups. Abundance correlation networks are shown, in which each circle (node) represents an operational taxonomic unit (OTU), its color, the bacterial phylum (blue, Firmicutes; green, Bacteroidetes; yellow, Actinobacteria; orange, Proteobacteria), and its size, the number of direct edges. Edges indicate the magnitude of distance correlation (positive in blue, negative in red). Only OTUs present in > 50% of the samples were taken into account, and only significant correlations (*p* < .05) are shown. The networks' parameters are presented in the table. The relative connectedness of the networks was calculated as the ratio between the number of significant interactions (edges) and the number of taxa (nodes) in the network. (b) Number of neighbors; means and standard error of mean (*SEM*) are indicated. **** indicates *p* < .0001

We built a bacterial abundance correlation network to assess the bacterial ecosystem structure. Compared with the CDE group, the HC showed a rich and complex network of correlations between bacteria as indicated by increased relative connectedness and fewer neighbors (Figure 3a,b).

**FIGURE 4:**
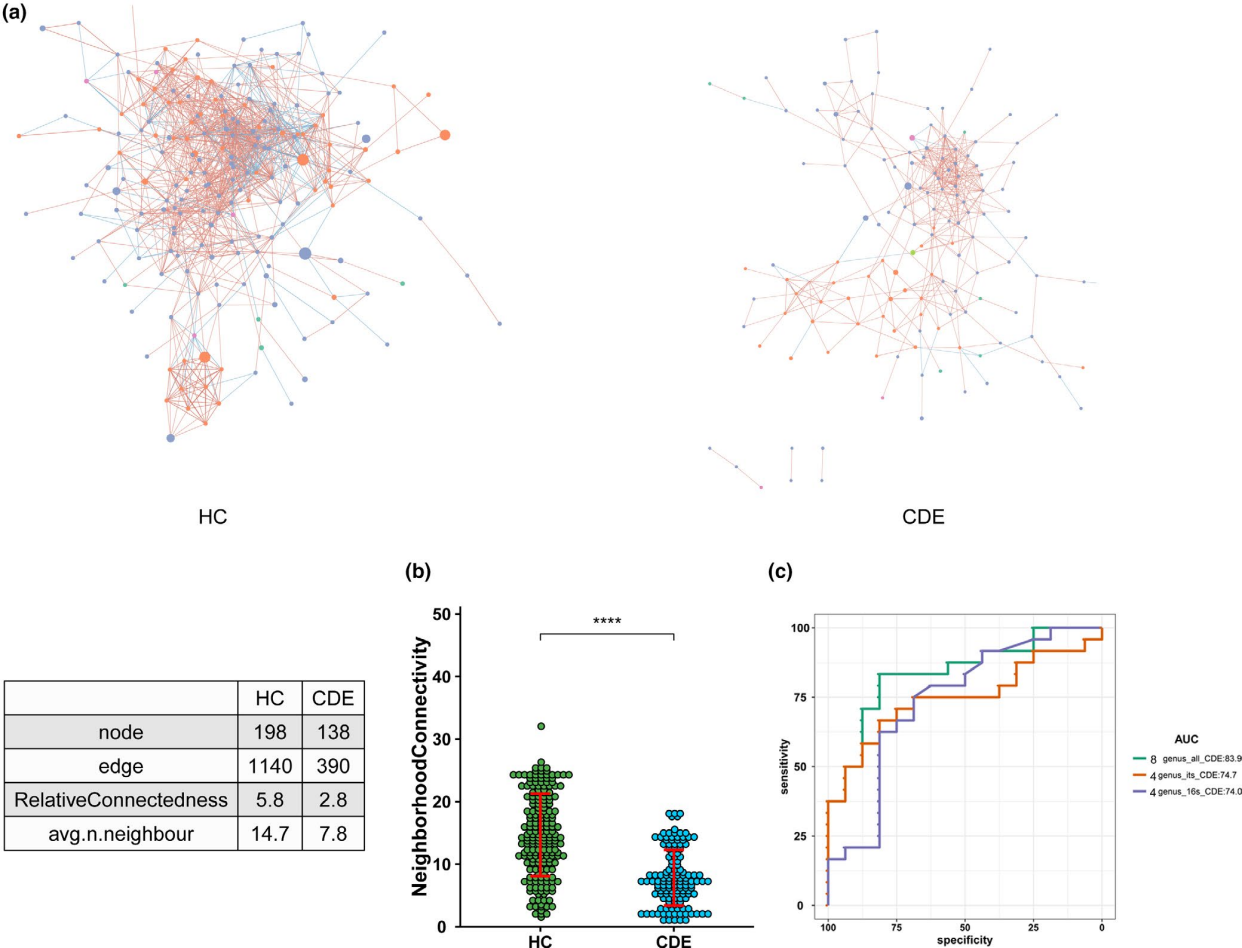
(a) Correlation networks at the family and genus levels were built in the two groups using distance correlation test. Abundance correlation networks are shown, in which each node represents a family or a genus, its shape, the kingdom (square, fungi; circle, bacteria), its color, and its size, the number of direct edges. Edges indicate the magnitude of distance correlation (positive in blue, negative in red). Only family and genus present in > 20% of the samples were taken into account, and only significant correlations (*p* < .05) are shown. The networks' parameters are presented in the table. The relative connectedness of the networks was calculated as the ratio between the number of significant interactions (edges) and the number of taxa (nodes) in the network. (b) Number of neighbors. Number of neighbors; means and *SEM* are indicated. **** indicates *p* < .0001. (c) Classification performance of multivariable logistic regression model using relative abundance of CDE‐associated genera was assessed by area under the receiving operational curve (ROC)

To further investigate the bacterial and fungi microbiota interactions according to disease phenotype, we built an abundance correlation network at the family and genus levels according to group. The interkingdom network was disrupted in the patients with CDE, as indicated by a decrease in the number of nodes and edges compared with the HC (Figure 4a,b).

### 3.5 Gut microbiome‐based signature discriminated disease status

We assessed the potential value of using the gut microbiota as biomarkers (Figure 4c) First, we found that the combination of four bacterial genera, *Akkermansia*, *Clostridium_sensu_stricto_1*, *UBA1819*, and *Veillonella*, could discriminate the CDE patients from the HC with an area under the curve (AUC) of 0.74 (95% confidence interval [CI] 0.57–0.91). Additionally, the combination of four fungal genera, *Candida*, *Chaetomium*, *Neocosmospora*, and *Occultifur*, distinguished between the CDE group and HC (AUC, 0.75 [95% CI: 0.59–0.9]). Moreover, combining these eight genera significantly improved the predictive performance (AUC, 0.84, [95% CI: 0.71–0.95]).

## 4 DISCUSSION

Our findings of differences in the composition of the gut mycobiota and altered bacterial–fungal interactions in the fecal samples of patients with CDE open a new avenue of research in gut microbiome dysbiosis in this population.

Previous investigations of the role of bacterial microbial communities in the gut of patients with CDE confirmed the presence of dysbiosis (Coello et al., 2019; Jiang et al., 2015). Our findings that the composition of the bacterial microbiota was altered in patients with major depression (Jiang et al., 2015) and BD (Jiang et al., 2019), as indicated by a decrease in the proportion of Dialister and Lachnospiraceae genera and an increase in the proportion of Acidaminococcaceae, are consistent with those of previous studies. The consistently low abundance of short‐chain fatty acid‐producing genera (Dialister and Lachnospiraceae) found in several previous studies (Jiang et al., 2015, 2018) suggests that a leaky gut is involved in the pathogenesis of CDE via bacterial translocation through the impaired intestinal barrier (Kiecolt‐Glaser et al., 2018; Maes, Kubera, Leunis, & Berk, 2012; Slyepchenko et al., 2017). The finding of high levels of Acidaminococcaceae in the feces of patients with depression (Jiang et al., 2015) and irritable bowel syndrome (Jeffery et al., 2012) has led to speculation that it may be related to intestinal symptoms. Further research is needed to clarify the role of this family in bowel function in CDE. Interestingly, we explored the enrichment in Akkermansia in patients with CDE. However, Flowers et al. found that Akkermansia levels were lower in obese than in nonobese patients with BD (Flowers, Evans, Ward, McInnis, & Ellingrod, 2017). Moreover, the authors found that antipsychotic medication was associated with a lower relative abundance of Akkermansia. Akkermansia is a monotypic genus in the branched phylum Verrucomicrobia, with Akkermansia muciniphila as its only species (Jayachandran, Chung, & Xu, 2019). The casual relationship between A. muciniphila and obesity has been confirmed in preclinical animal studies (Anhe et al., 2015; Dao et al., 2016). The association of antipsychotic medication with weight gain, the accumulation of adipose tissue, and metabolic side effects is well established (Bretler, Weisberg, Koren, & Neuman, 2019; Li et al., 2013). Although most of our patients were receiving antipsychotic treatment, only those with a normal BMI were included in the study, leading us to speculate that the higher levels of Akkermansia in these patients may have prevented antipsychotic‐associated obesity.

Prompted by recent studies of the gut fungal community in patients with autism (Strati et al., 2017) and Rett syndrome (Strati et al., 2016), we performed a sequence analysis of the ITS1 marker gene to profile the fungal microbiome in patients with CDE. Ours is the first report of fungal dysbiosis in patients with CDE characterized by altered biodiversity and a changed composition. We found that fecal fungal diversity (estimated using the ACE and Chao indexes) was lower in patients with CDE, in contrast to findings in patients with neurodevelopmental conditions showing no significant differences in alpha diversity indexes (Strati et al., 2017). However, our findings are consistent with those of previous studies showing a relative decrease in biodiversity in patients with irritable bowel symptom (Botschuijver et al., 2017) and inflammatory bowel diseases (Qiu et al., 2017), which have a high rate of comorbidity with anxiety and depressive disorder (Hanlon, Hewitt, Bell, Phillips, & Mikocka‐Walus, 2018).

The relative abundance of Candida (phylum Ascomycota) was higher in the CDE patients than in the HC. Strati et al. reported overgrowth of Candida in patients with autism (Strati et al., 2017) and Rett syndrome (Strati et al., 2016). *Candida albicans*, the main species of the *Candida* genus, is thought to be a resident pathogenic commensal in the gut, which can translocate into the mesenteric lymph nodes or systemic circulation due to increased permeability of the gut wall in individuals exposed to stress (Mamouei, Zeng, Wang, & Wang, 2017). Consistent with this, Severance et al. quantified *C. albicans* IgG antibodies in patients with schizophrenia and BD and found that elevated IgG levels were associated with gastrointestinal symptoms. Furthermore, the authors found that probiotics normalized *C. albicans* antibody levels and *C. albicans*‐associated gut discomfort in many patients with schizophrenia (Severance et al., 2016). Although several hypotheses have been proposed to explain the involvement of *C. albicans* in CDE, the underlying mechanisms remain speculative. One hypothesis postulates that the mucocutaneous pathobiont *C. albicans* is the major direct inducer of human antifungal Th17 cells (Shao et al., 2019). Thus, the overgrowth of Candida in the gut leads to an increase in the proportion of gut Th17 cells, which, after entering the peripheral circulation, penetrate the blood‐brain barrier (BBB) and migrate into the brain where they activate the hippocampal microglia and induce depressive behaviors (Slyepchenko et al., 2016). Second, pathogenic *Candida* spp. in the gastrointestinal tract gradually release toxins, such as gliotoxin, into the bloodstream. After crossing the BBB, these toxins may target central nervous system astrocytes, which are integral in maintaining the integrity of the BBB, and oligodendrocytes (Purzycki & Shain, 2010). Third, higher *Candida* levels in the gut may reflect bacterial dysbiosis, which has been shown to induce depressive‐like behavior in fecal transplantation studies.

Fungi and bacteria coexist and interact in the human and animal gut. Moreover, the relationship may be competitive; for example, antibiotic‐induced depletion of gut bacteria facilitates the expansion of fungi, particularly in the gut (Noverr, Noggle, Toews, & Huffnagle, 2004). Furthermore, germ‐free mice are susceptible to infection with fungi such as Candida (Dollive et al., 2013). The commensal bacteria Bacteroides thetaiotaomicron and Blautia producta can reduce Candida colonization via secretion of antifungal peptides from colonic epithelial cells (Fan et al., 2015). In addition to alterations in fungal diversity, we noted a disease‐specific pattern in the interkingdom network such that the correlation network between bacteria and fungi was disrupted in patients with CDE. Our study is the first to describe gut bacterial–fungal interactions associated with psychiatric conditions. Sovran et al. (2018) reported that Enterobacteriaceae cooperate with fungi to favor their colonization and an active role in intestinal inflammation in an animal model of colitis. Given the immune pathway in the gut microbiota–brain axis, it is reasonable to hypothesize that altered bacterial and fungal biodiversity results in new interkingdom interactions that may be involved in the inflammatory process. Further study is needed to explore the functional connections between fungi and bacteria in the gut.

The main limitations of our study are the small sample size and cross‐sectional design, which did not allow us to establish a casual relationship. A longitudinal study is needed to confirm whether the observed relationships between specific gut mycobiota genera and depressive symptoms persist over time. Furthermore, lifestyle and behavioral confounders are always a concern in microbiota research. Given the lack of information on diet, any variations observed could theoretically be the result of a diet bias. Thus, further studies that consider the diet habit are needed to clarify this association. Finally, while the fungal composition is significantly different between the intestinal mucosa and stool, an investigation of the mucosal mycobiota is beyond the scope of this study.

In conclusion, we found that CDE is characterized by an altered fungal gut microbiota associated with a disruption in the fungi‐bacteria correlation network, suggesting that interkingdom interactions between bacteria and fungi are involved in the development of CDE. While the implications remain to be refined, our findings provide novel insight into the development of mycobiota‐based biomarkers and the treatment of CDE. Future studies involving larger cohorts with metagenomic or metabolomics analyses are needed to further elucidate the impact of the bacterial and fungal microbiota on the development of CDE.

## CONFLICTS OF INTEREST

The authors declare that they have no competing interests. The funders had no role in study design, data collection, and analysis, decision to publish, or preparation of the manuscript.

## AUTHORS’ CONTRIBUTIONS

H.Y.J. and B.R. conceived the study and revised the manuscript critically for important intellectual content. H.Y.J., X.Z., and L.Y.P. made substantial contributions to its design, acquisition, analysis, and interpretation of data. Z.Z. and Y.Y.Z. participated in the design, acquisition, analysis, and interpretation of data. All authors read and approved the final manuscript.

## Supporting information

Supplementary Material

## Data Availability

The data that support the findings of this study are available from the corresponding author upon reasonable request.
