# Supplementary material for: Altered gut bacterial–fungal interkingdom networks in patients with current depressive episode
Source: Brain Behav. 2020 Jun 12;10(8):e01677. doi: 10.1002/brb3.1677 (PMC7428472; doi:10.1002/brb3.1677)

| **Table S1** Bacterial α-diversity comparison in patients with CDE | | | | | | | | | | | |
| --- | --- | --- | --- | --- | --- | --- | --- | --- | --- | --- | --- |
|  | BD | MDD | P |  | Antidepressant | No-antidepressant | P |  | Antipsychotic | No-antipsychotic | P |
| Sobs | 239.2±59.05 | 225.36±55.37 | 0.63 |  | 226.6±66.8 | 230.6±45.8 | 0.81 |  | 218.6±51.6 | 236.9±58.3 | 0.33 |
| Shannon | 3.33±0.38 | 3.08±0.62 | 0.39 |  | 3.2±0.62 | 3.23±0.37 | 0.97 |  | 3.1±0.58 | 3.25±0.54 | 0.72 |
| Simpson | 0.092±0.07 | 0.13±0.08 | 0.3 |  | 0.1±0.05 | 0.1±0.06 | 0.97 |  | 0.12±0.09 | 0.1±0.07 | 0.63 |
| Ace | 280.88±74.1 | 276.05±63.1 | 0.97 |  | 281.2±74.2 | 270.1±57.8 | 0.58 |  | 266.1±63 | 282.2±68.7 | 0.76 |
| Chao | 285.95±75.12 | 227.64±61.6 | 0.88 |  | 283.6±73.6 | 270.9±57.4 | 0.58 |  | 266.2±62.8 | 285.7±67.5 | 0.51 |

| **Table S2** Fungal α-diversity comparison in patients with CDE | | | | | | | | | | | |
| --- | --- | --- | --- | --- | --- | --- | --- | --- | --- | --- | --- |
|  | BD | MDD | P |  | Antidepressant | No-antidepressant | P |  | Antipsychotic | No-antipsychotic | P |
| Sobs | 64.1±44.4 | 66.4±28 | 0.68 |  | 66.6±40.7 | 64.3±29.8 | 1 |  | 60.4±16.4 | 68.4±42.7 | 0.95 |
| Shannon | 1.57±1.4 | 1.75±1.24 | 0.88 |  | 1.6±1.35 | 1.78±1.26 | 0.93 |  | 1.67±1.23 | 1.67±1.35 | 0.95 |
| Simpson | 0.5±0.39 | 0.43±0.35 | 0.83 |  | 0.5±0.36 | 0.41±0.37 | 0.83 |  | 0.47±0.38 | 0.45±0.37 | 0.95 |
| Ace | 70.8±42.5 | 77.3±28.3 | 0.42 |  | 73.7±39.9 | 75.6±29.1 | 0.75 |  | 71.46±20.18 | 76.5±41 | 0.9 |
| Chao | 69.4±42.7 | 72.1±27.8 | 0.88 |  | 70.4±40.5 | 71.5±27.7 | 1 |  | 66.1±16.2 | 73.9±41.5 | 0.95 |

**Figure S1** PCoA of unweighted UniFrac analysis demonstrated the bacterial microbiome composition of CDE was similar to HC.


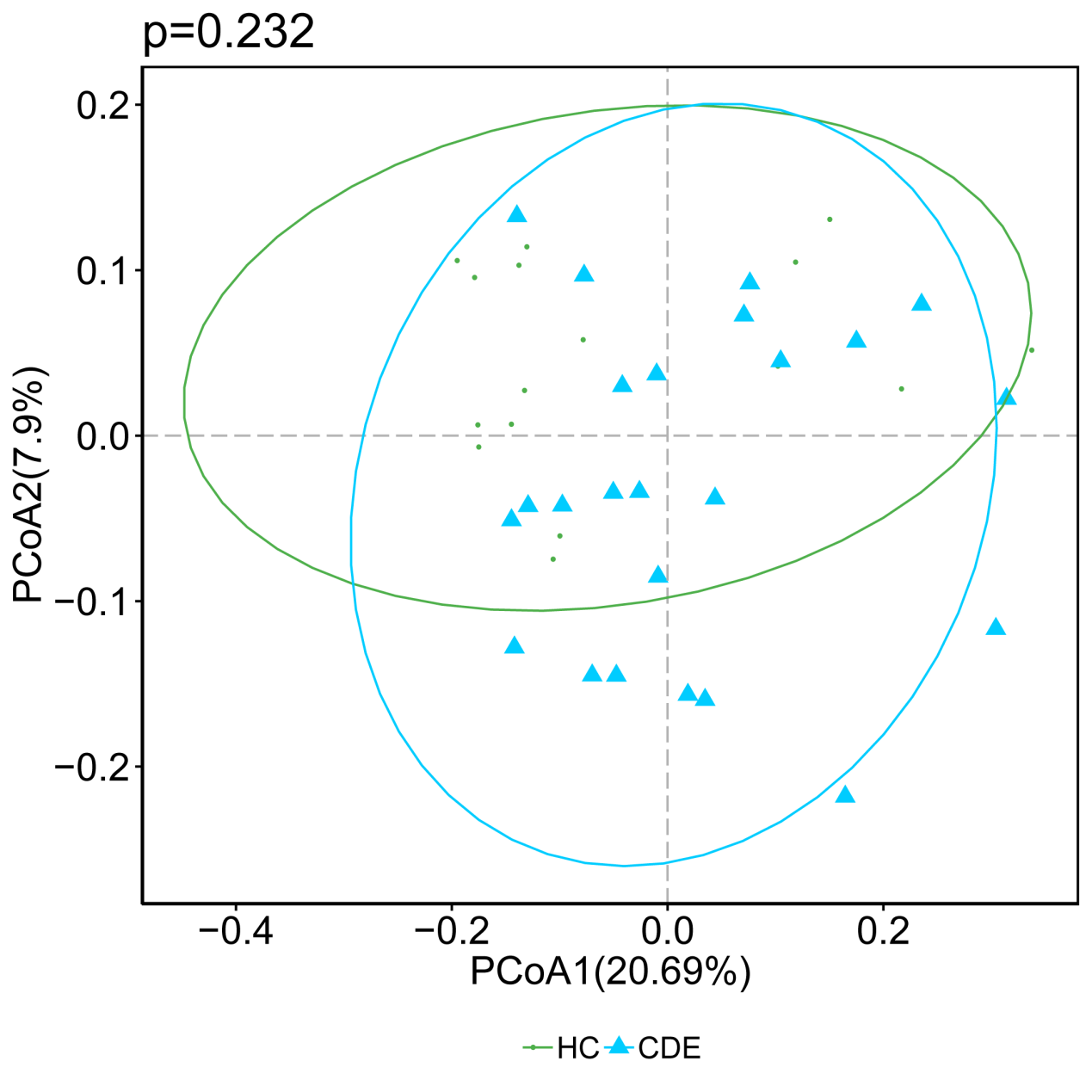


**Figure S2** PCoA of weighted UniFrac analysis demonstrated the bacterial microbiome composition of CDE was similar to HC.


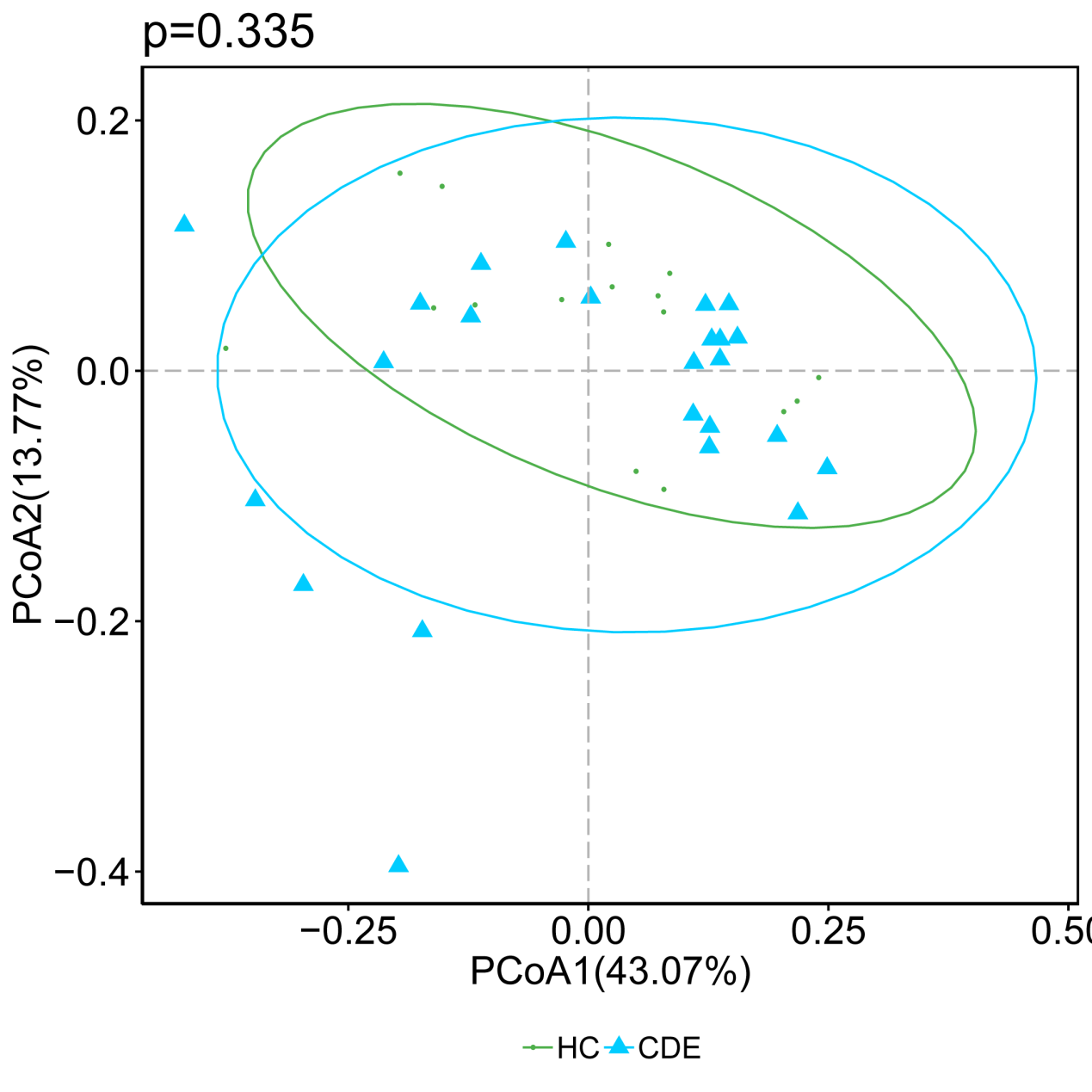


**Figure S3** PCoA of bray curtis analysis demonstrated the bacterial microbiome composition of MDD was similar to BD


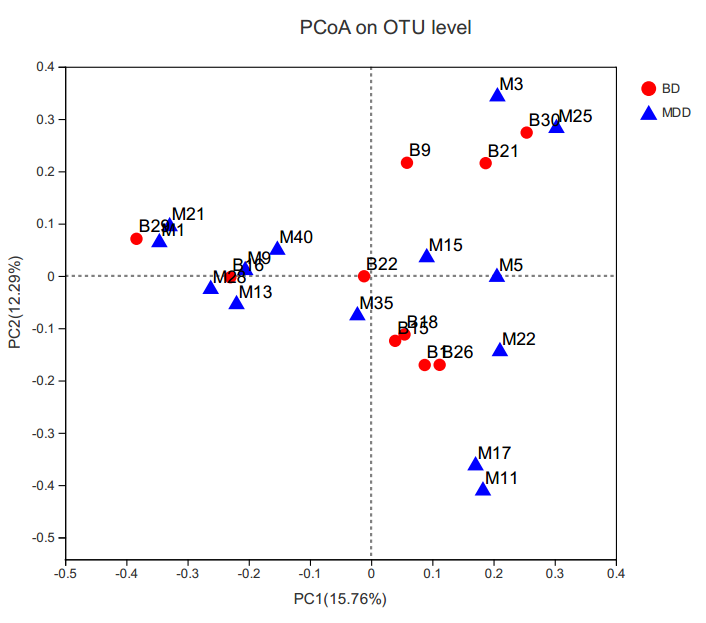
.

**Figure S4** PCoA of unweighted analysis demonstrated the bacterial microbiome composition of MDD was similar to BD


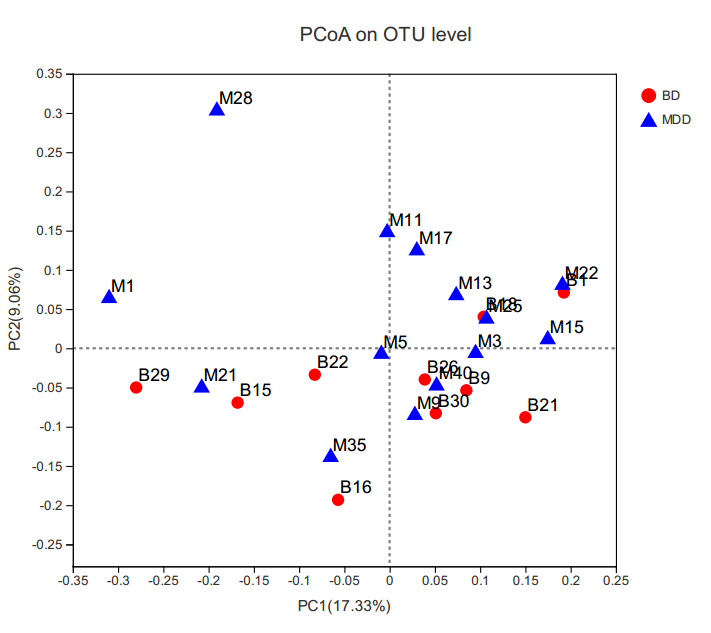


**Figure S5** PCoA of weighted analysis demonstrated the bacterial microbiome composition of MDD was similar to BD


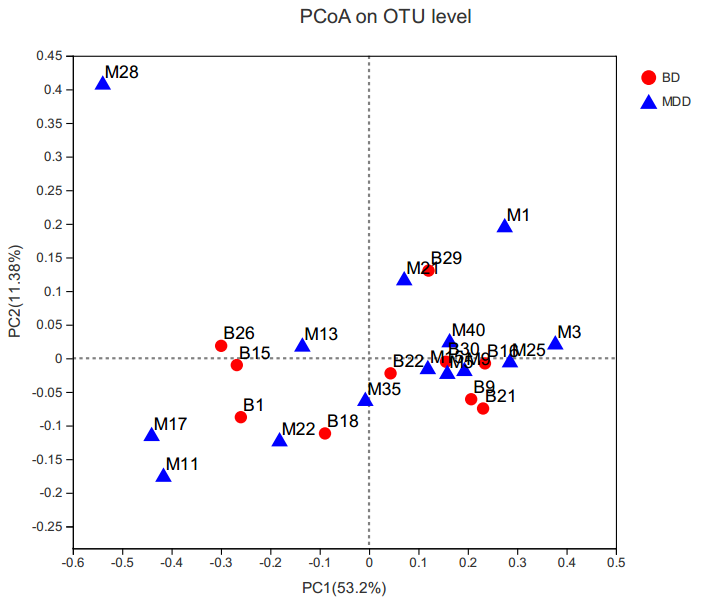


**Figure S6** PCoA of bray curtis analysis demonstrated the bacterial microbiome composition of antidepressant users and no-antidepressant users in CDE


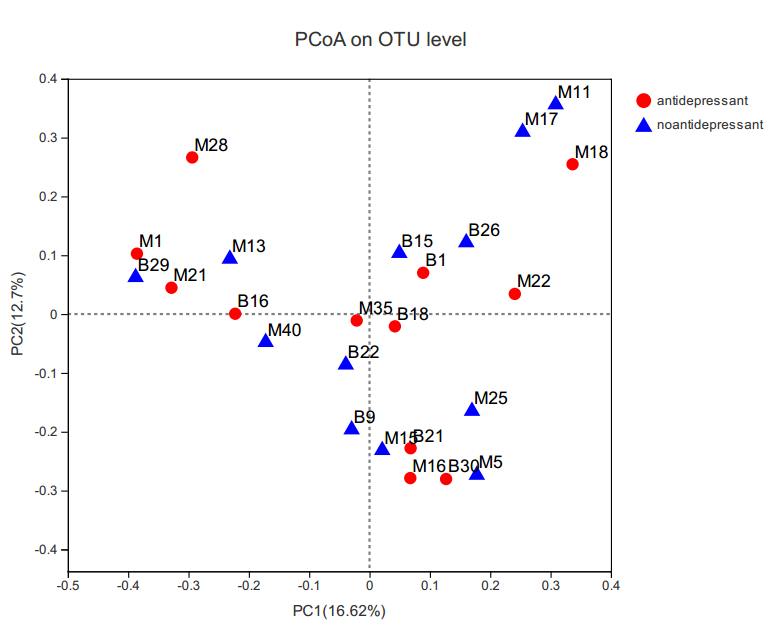


**Figure S7** PCoA of unweighted analysis demonstrated the bacterial microbiome composition of antidepressant users and no-antidepressant users in CDE


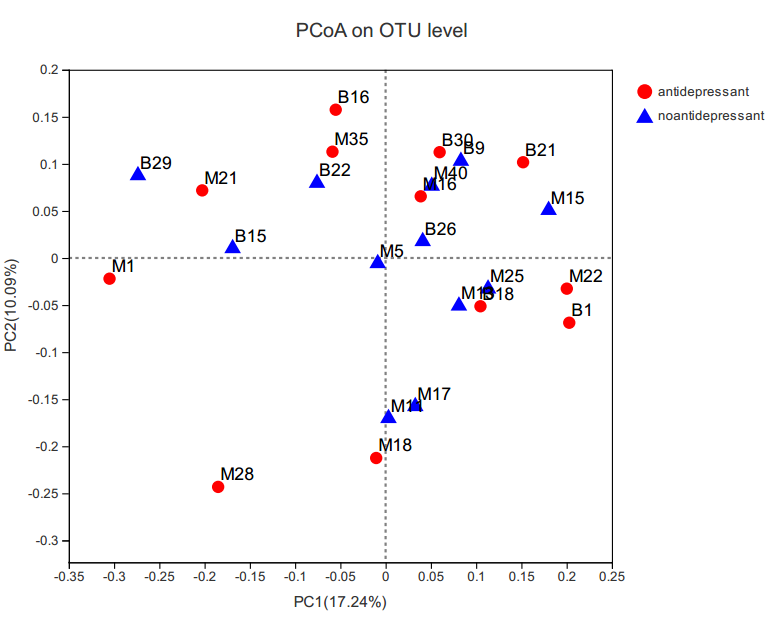


**Figure S8** PCoA of weighted analysis demonstrated the bacterial microbiome composition of antidepressant users and no-antidepressant users in CDE


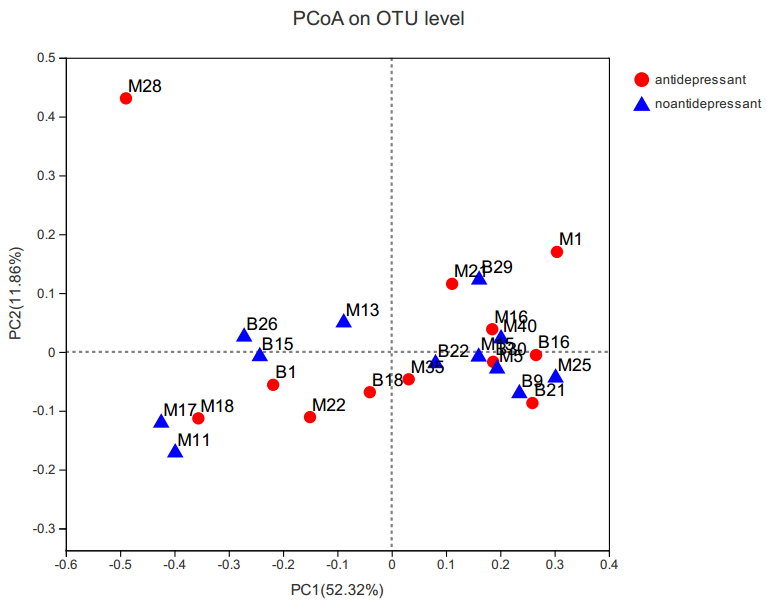


**Figure S9** PCoA of bray curtis analysis demonstrated the bacterial microbiome composition of antipsychotic users and no-antipsychotic users in CDE

**
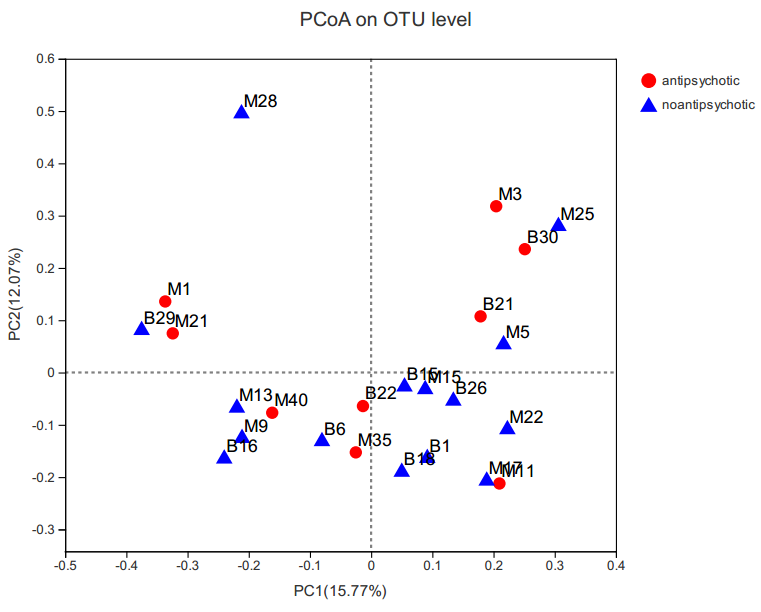
**

**Figure S10** PCoA of unweighted analysis demonstrated the bacterial microbiome composition of antipsychotic users and no-antipsychotic users in CDE

**
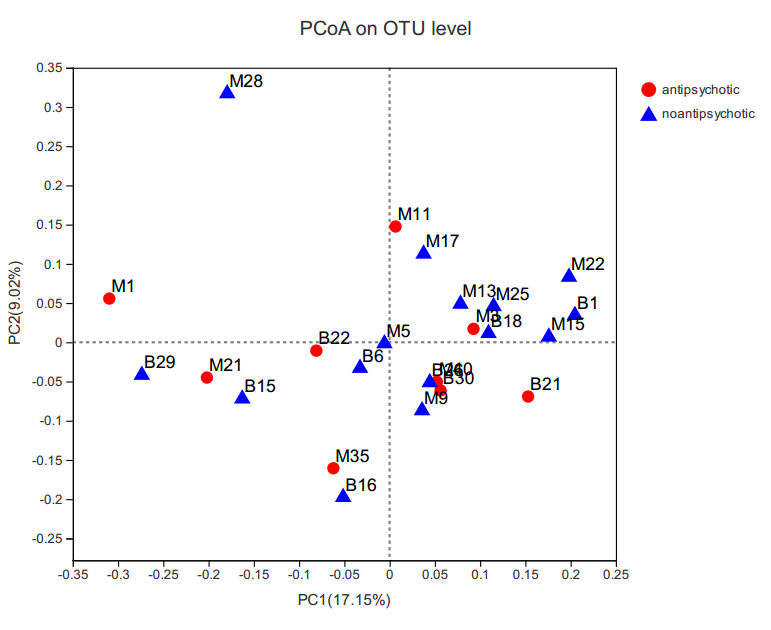
**

**Figure S11** PCoA of weighted analysis demonstrated the bacterial microbiome composition of antipsychotic users and no-antipsychotic users in CDE

**
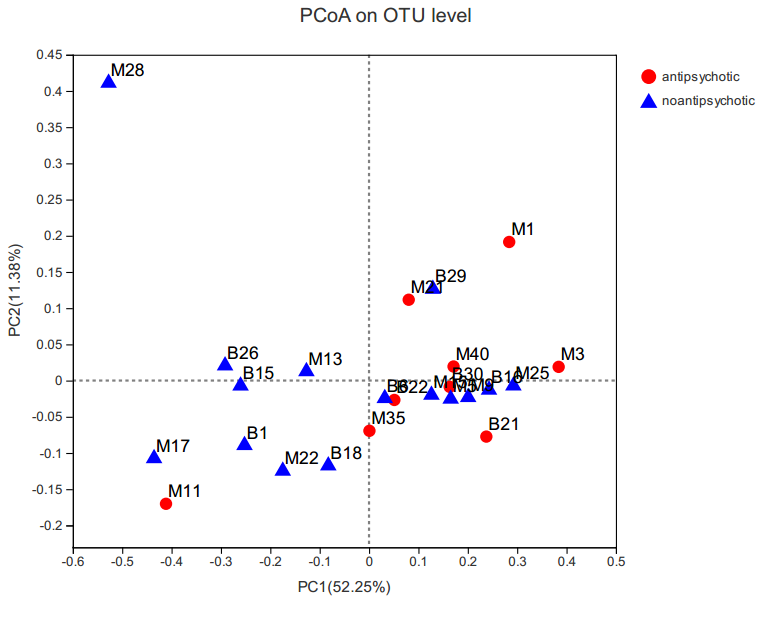
**

**Figure S12** PCoA of bray curtis analysis demonstrated the fungal microbiome composition of CDE was similar to HC.


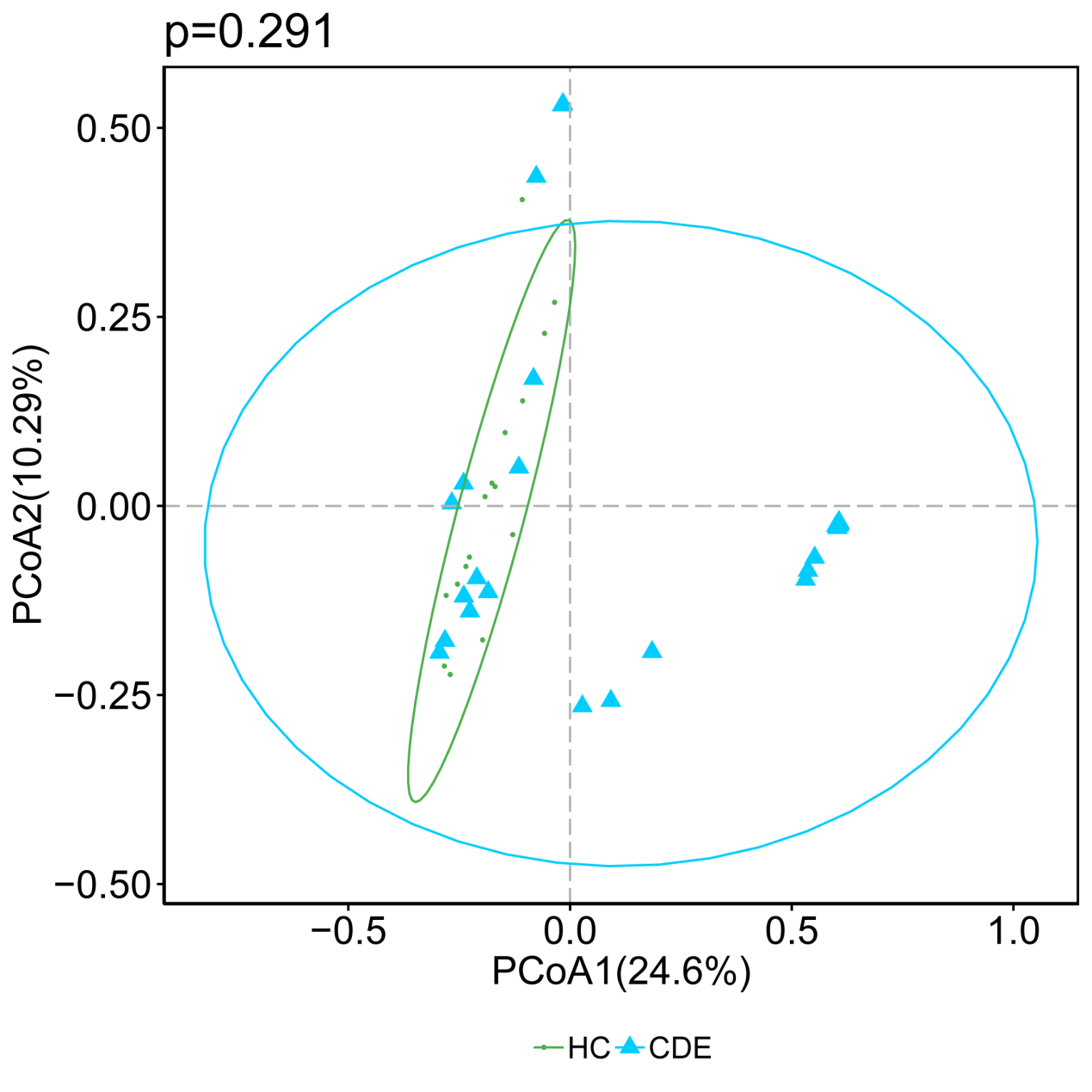


**Figure S13** PCoA of unweighted UniFrac analysis demonstrated the fungal microbiome composition of CDE was similar to HC.


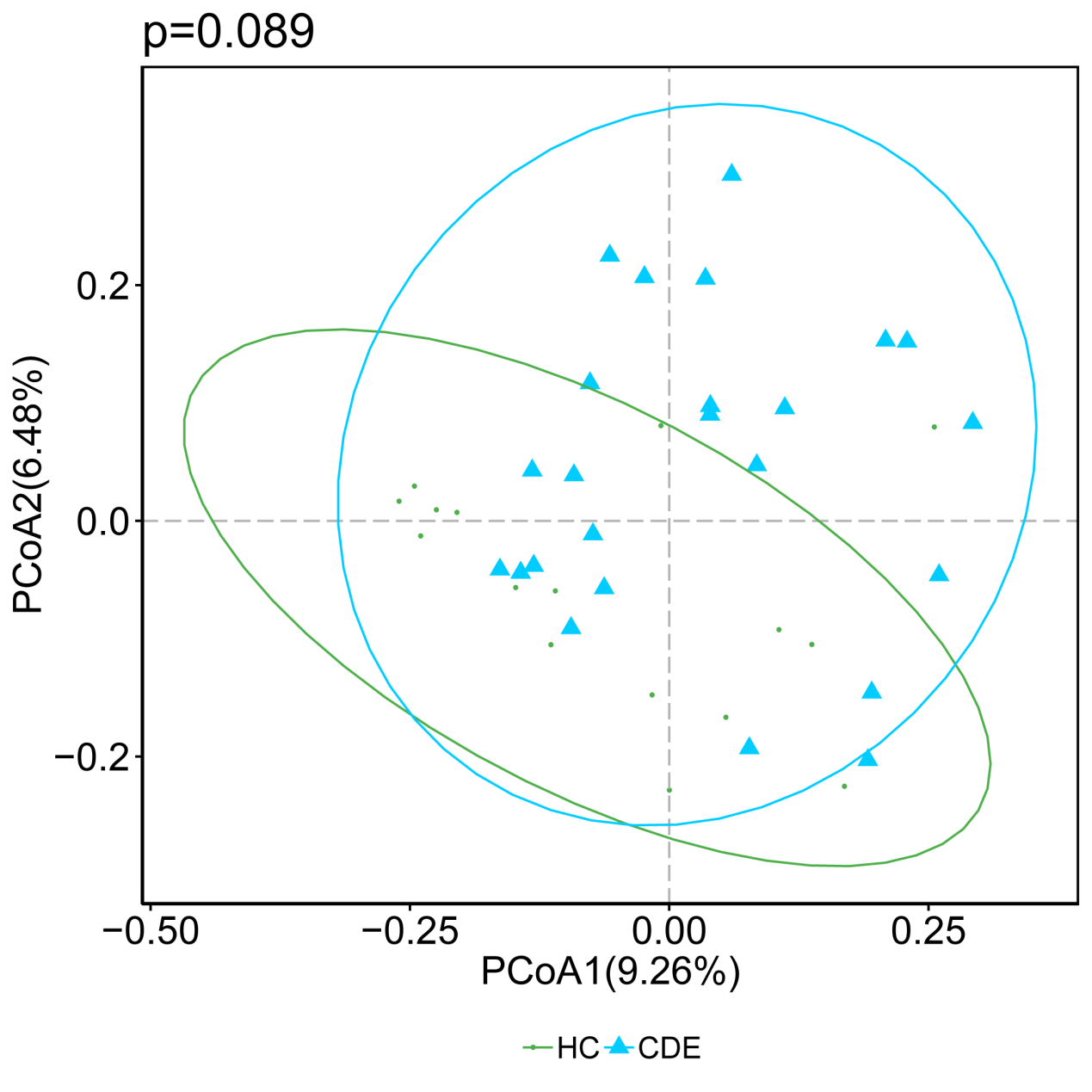


**Figure S14** PCoA of weighted UniFrac analysis demonstrated the fungal microbiome composition of CDE was similar to HC.


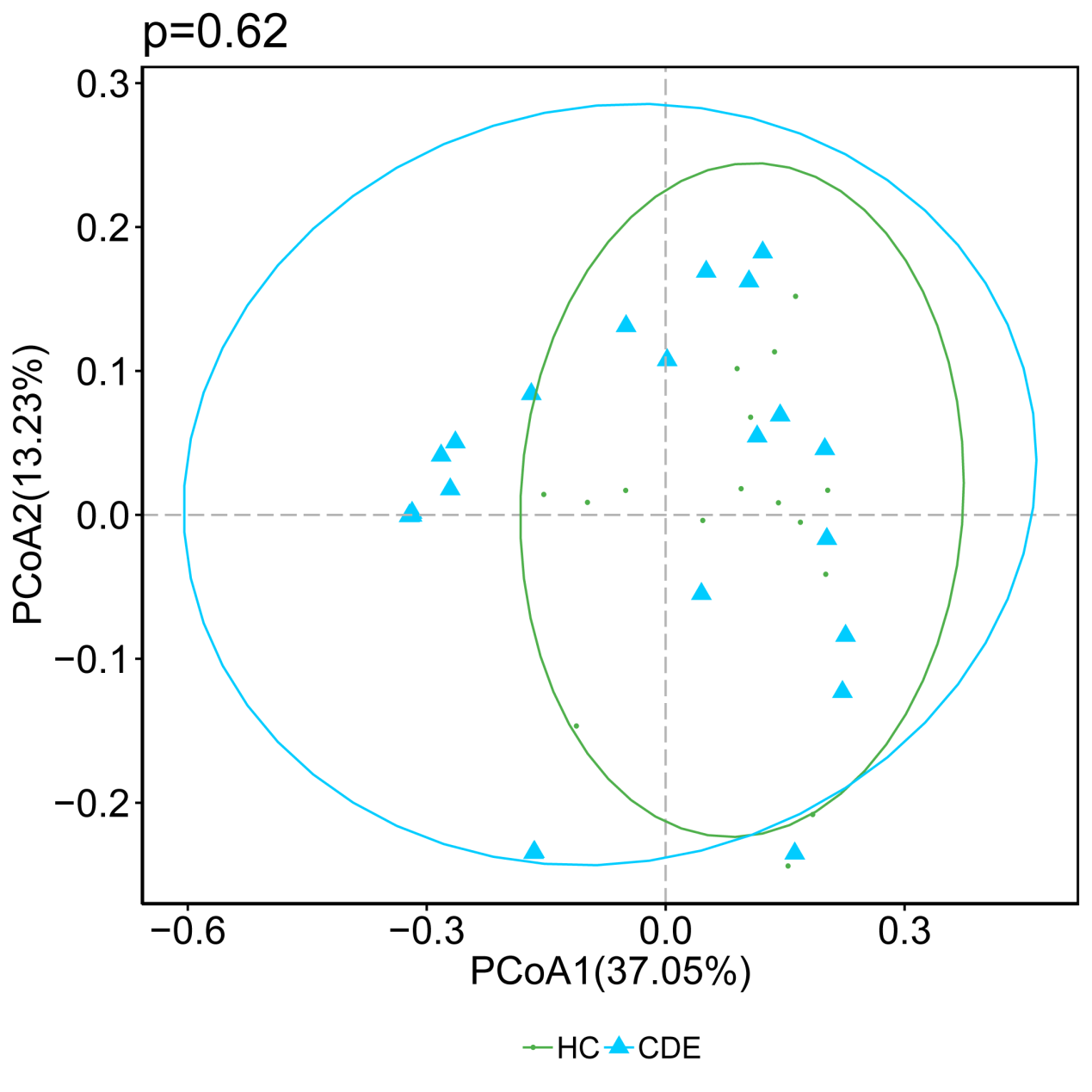


**Figure S15** PCoA of bray curtis analysis demonstrated the fungal microbiome composition of MDD was similar to BD


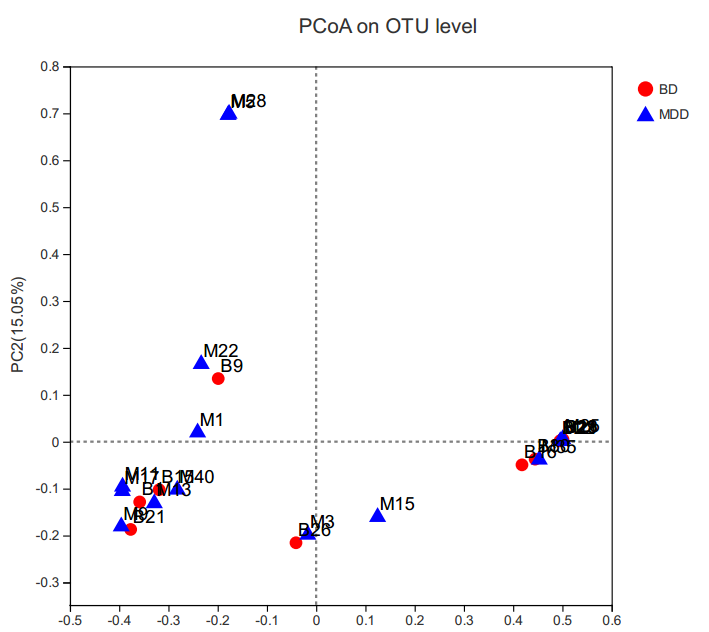


**Figure S16** PCoA of unweighted analysis demonstrated the fungal microbiome composition of MDD was similar to BD


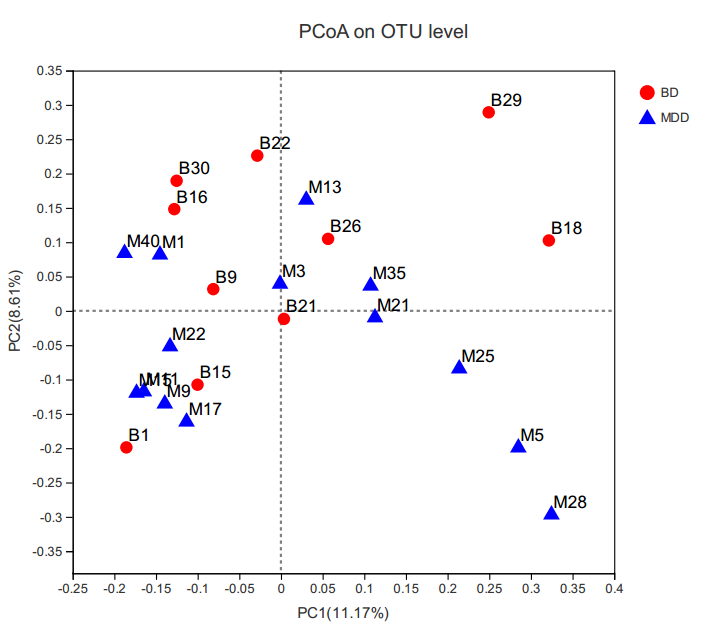


**Figure S17** PCoA of weighted analysis demonstrated the fungal microbiome composition of MDD was similar to BD


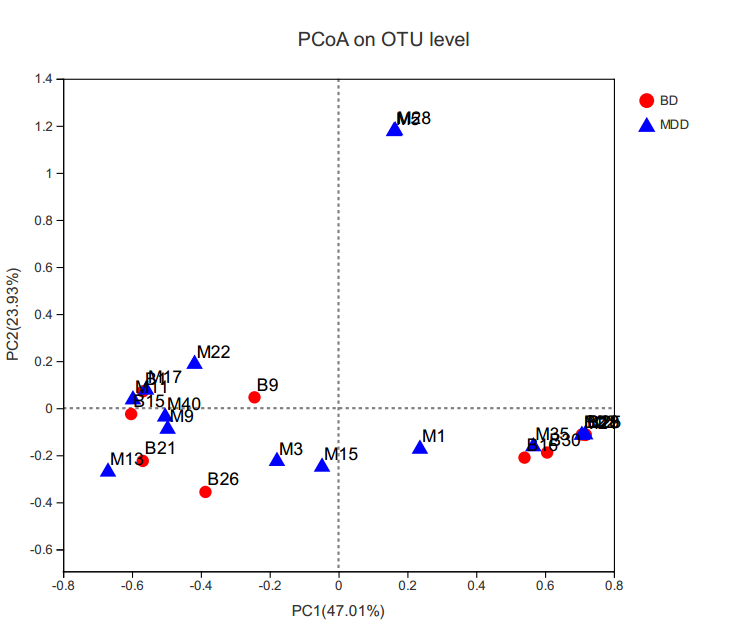


**Figure S18** PCoA of bray curtis analysis demonstrated the fungal microbiome composition of antidepressant users was similar to no-antidepressant users in CDE


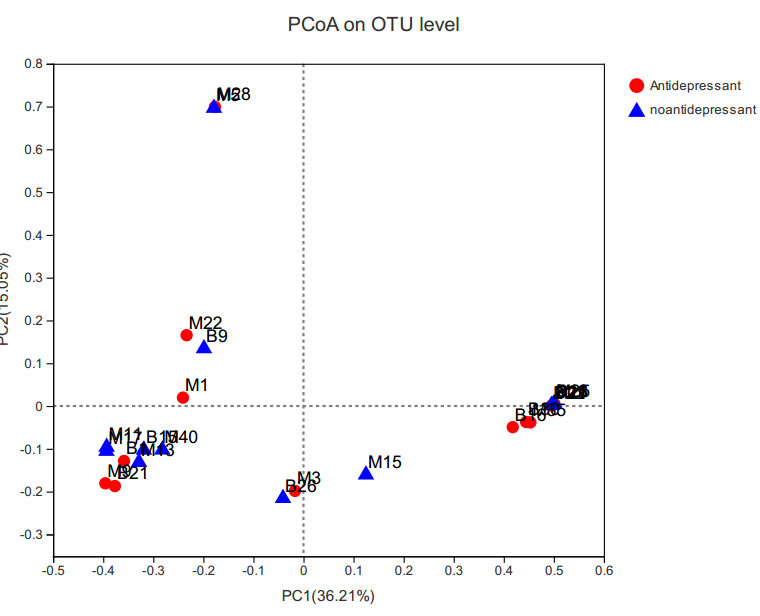


**Figure S19** PCoA of unweighted analysis demonstrated the fungal microbiome composition of antidepressant users was similar to no-antidepressant users in CDE


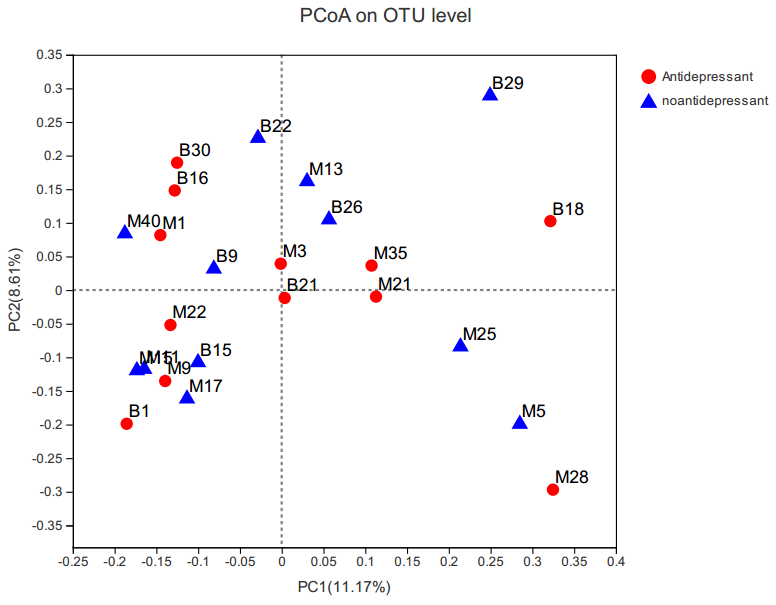


**Figure S20** PCoA of weighted analysis demonstrated the fungal microbiome composition of antidepressant users was similar to no-antidepressant users in CDE


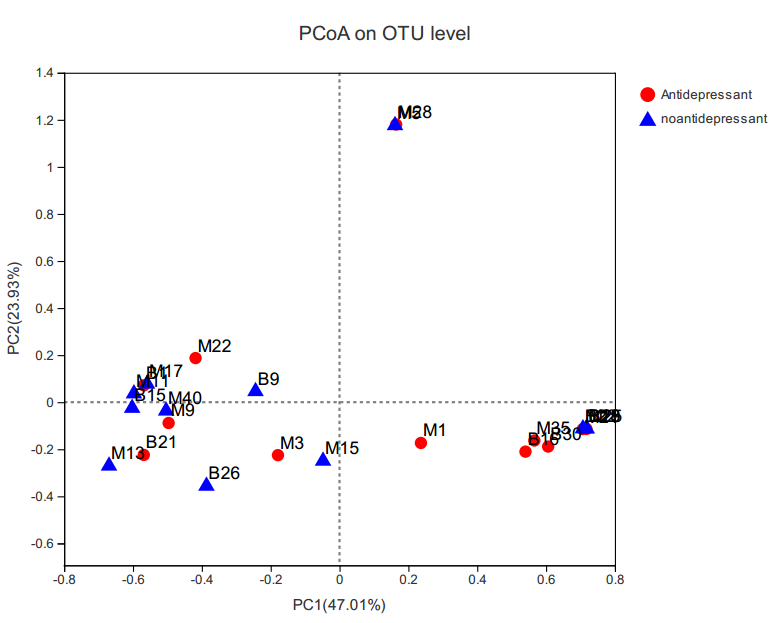


**Figure S21** PCoA of bray curtis analysis demonstrated the fungal microbiome composition of antipsychotic users was similar to no-antipsychotic users in CDE


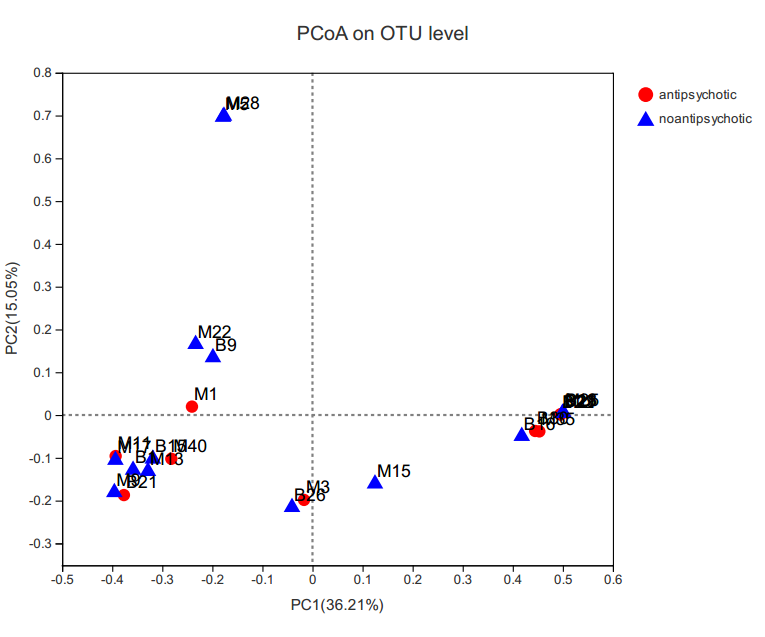


**Figure S22** PCoA of unweighted analysis demonstrated the fungal microbiome composition of antipsychotic users was similar to no-antipsychotic users in CDE


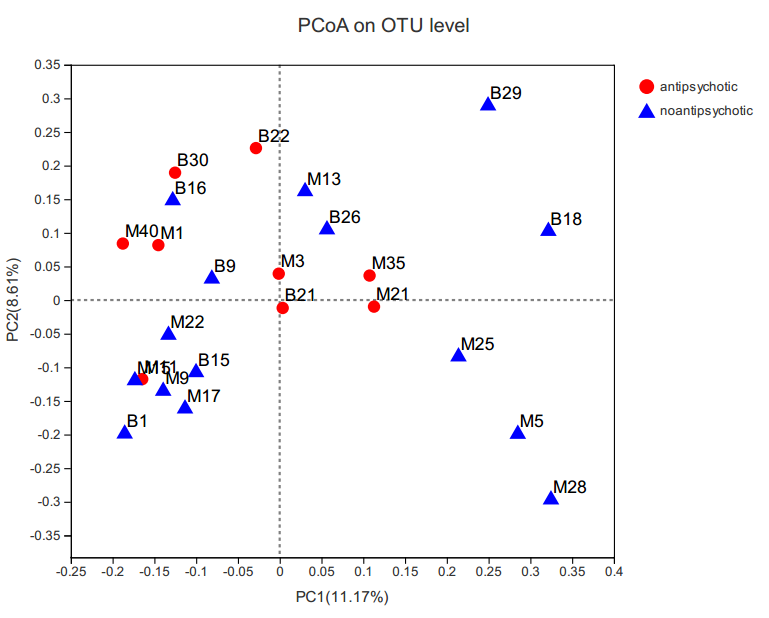


**Figure S23** PCoA of weighted analysis demonstrated the fungal microbiome composition of antipsychotic users was similar to no-antipsychotict users in CDE


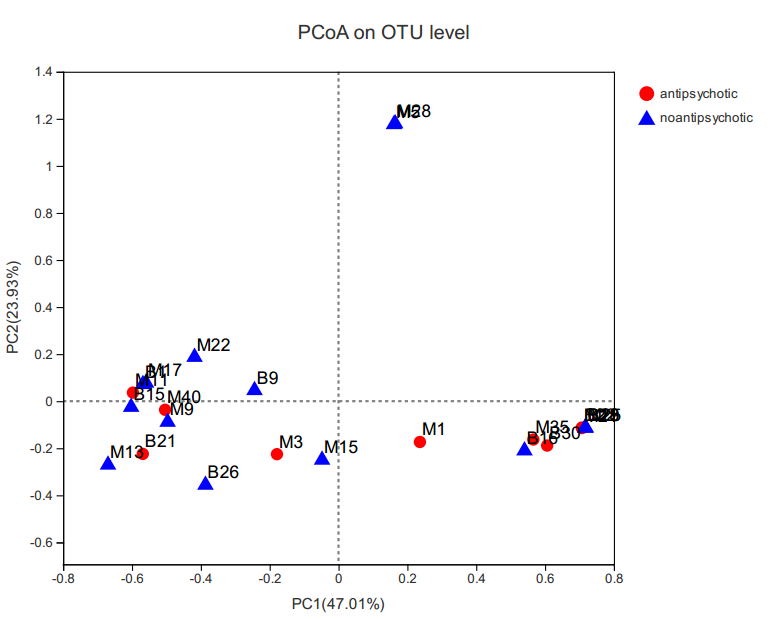

Supplement: Supplementary file 1 — Supplementary Material [file BRB3-10-e01677-s001.docx]
